# Blistering Behavior of Beryllium and Beryllium Alloy under High-Dose Helium Ion Irradiation

**DOI:** 10.3390/ma17163997

**Published:** 2024-08-11

**Authors:** Ping-Ping Liu, Qi-Cong Wang, Yu-Mei Jia, Wen-Tuo Han, Xiao-Ou Yi, Qian Zhan, Fa-Rong Wan

**Affiliations:** 1 School of Materials Science and Engineering, University of Science and Technology Beijing, Beijing 100083, China; 13964134761@139.com (Q.-C.W.);; 2 USTB-BJHB Joint Laboratory of Beryllium and Advanced Materials for Fusion Energy, University of Science and Technology Beijing, Beijing 100083, China

**Keywords:** fusion energy, neutron multiplier, blister, swelling, Be and Be_12_M (Ti, W) pebble, internal pressure of bubble

## Abstract

Beryllium (Be) has been selected as the solid neutron multiplier material for a tritium breeding blanket module in ITER, which is also the primary option of the Chinese TBM program. But the irradiation swelling of beryllium is severe under high temperature, high irradiation damage and high doses of transmutation-induced helium. Advanced neutron multipliers with high stability at high temperature are desired for the demonstration power plant (DEMO) reactors and the China Fusion Engineering Test Reactor (CFETR). Beryllium alloys mainly composed of Be_12_M (M is W or Ti) phase were fabricated by HIP, which has a high melting point and high beryllium content. Beryllium and beryllide (Be_12_Ti and Be_12_W) samples were irradiated by helium ion with 30 keV and 1 × 10^18^ cm^−2^ at RT. The microstructures of Be, Be_12_Ti and Be_12_W samples were analyzed by SEM and TEM before and after ion irradiation. The average size of the first blistering on the surface of Be-W alloy is about 0.8 μm, and that of secondary blistering is about 79 nm. The surface blistering rates of the beryllium and beryllide samples were also compared. These results may provide a preliminary experimental basis for evaluating the irradiation swelling resistance of beryllium alloy.

## 1. Introduction

Amid the global energy crisis and worsening climate emergency, we urgently need a carbon-free, safe, clean and limitless energy source. Fusion energy has the potential to meet this need. The idea and design of a controlled fusion reactor dates back to the 1950s [1,2]. United States astrophysicist L. Spitzer at Princeton University [1] and O. A. Lavrent’ev [2], a Red Army soldier from the Soviet Union, respectively, proposed a method of achieving controlled nuclear fusion. The story of O. A. Lavrent’ev is very legendary, but little known was known about it for reasons of secrecy [2]. In 1958, at the Second International Conference on the Peaceful Uses of Atomic Energy held in Geneva, Switzerland, Great Britain, the United States and the Soviet Union reached an agreement, and since then, controlled nuclear fusion research has entered the stage of international cooperation. The closest piece of fusion history belongs to the International Thermonuclear Experimental Reactor (ITER) project. ITER and future commercial power demonstration reactors (DEMO) have been designed and developed by scientists around the world [3,4]. However, one key problem—“tritium fuel self-sustaining”—needs to be solved first. Thus, the tritium breeding blanket module (TBM) was developed.

Beryllium (Be) is a natural neutron multiplier material. It can react with a neutron, with energy over 1.9 MeV, a (n, 2n) reaction, to produce two alpha particles and two neutrons. Beryllium pebbles are selected to be used as neutron multipliers in the Helium-cooled solid ceramic breeder (HCCB) test TBM of ITER, which is also chosen by the Chinese TBM program [5,6,7]. However, the beryllium pebbles will undergo severe irradiation swelling processes under high temperature, high irradiation damage and high doses of transmutation-induced helium in the China Fusion Engineering Test Reactor (CFETR) and the future demonstration fusion power plant (DEMO) [8,9], mainly because the melting point of beryllium is low (1558 K) and the starting temperature of swelling by neutron irradiation is low (~773 K). For example, the irradiation swelling rate of beryllium pebbles is >8% under neutron irradiation (~37 dpa and ~6000 appm He) at about 873 K [10,11]. Therefore, beryllium intermetallic compounds that have high melting point have been expected as the advanced neutron multiplier material for fusion CFETR and DEMO blanket [12,13].

Recently, Hwang et al. [14] reported that a ternary beryllide was successfully prepared by mixing Be_12_V with Be_12_Ti and using plasma sintering. They also evaluated the mechanical properties of beryllide (Be_12_Ti) fabricated using plasma sintering and revealed that the tensile strengths increased with increasing sintering temperature [15,16]. Gaisin et al. [17] had reported the production of Ø90 mm × 90 mm tantalum beryllide by Ulba, as well as Ø200 mm × 200 mm titanium beryllide and chromium beryllide samples, with the density of the beryllide preforms exceeding 96%, demonstrating their high quality and potential for various applications. Kawamura et al. [18] reports the fabrication of Be_12_Ti (the melting point is about 1823 K) and a compatibility test between Be_12_Ti and SS316LN. The Be_12_Ti specimens were fabricated by the hot isostatic pressing (HIP) process from beryllium and titanium powder by NGK Insulators Ltd. (Nagoya, Japan). The results show that the thickness of the reaction layer between Be_12_Ti and SS316LN was much smaller than that between Be and SS316LN at high temperature (873–1073 K). Iwakiri et al. [19] examined the deuterium retention and desorption properties of Be_12_Ti by using D ion implantation. Total retention of deuterium in Be_12_Ti is much smaller than that of beryllium over a wide temperature range from RT to 873 K. Munakata et al. [20] reports that Be_12_Ti is less reactive with water vapor in comparison with beryllium. Zimber et al. [21] analyzed the absorption site of hydrogen and helium in beryllium and presented novel insights into beryllium–hydrogen interaction behavior. Chakin et al. [22,23,24] investigated tritium release and retention in beryllium and titanium beryllide after neutron irradiation. The microstructure of Be-7Ti, which was manufactured by vacuum melting and vacuum casting, contains two phases, mainly, Be_12_Ti, and also a small amount of Be. However, the sample from Brush Wellman Inc has only a single-phase Be_12_Ti. The thermal-programmed desorption tests showed a much lower tritium retention in Be-7Ti than in Be from 746 K to 1040 K. Nakamichi et al. [25,26,27] developed a plasma sintering method of Be-Ti alloy and obtained a single phase of Be12Ti by homogenization treatment. However, the homogenized Be_12_Ti pebbles tend to be more reactive with water vapor than that of pebbles not homogenized, because homogenization causes many micropores, increasing the specific surface area. To prevent this reactivity, they fabricated prototypic pebbles with Be_12_V composition [27]. Kim et al. [28] fabricated binary (BeTi, BeV) and ternary (BeTiV) beryllium alloy pebbles by combining plasma sintering and rotating electrode methods. Deuterium desorption and retention properties were investigated by D ion implantation and thermal desorption spectroscopy. The results indicated that the beryllide pebbles exhibited better deuterium desorption properties, such as lower initial release temperature, total retention, and activation energy for deuterium desorption than beryllium [28]. However, the irradiation swelling and even irradiation blistering behavior of the beryllide (Beryllium alloy) require investigation to provide experimental data and reference for the feasibility evaluation of DEMO advanced neutron multiplier material.

In this work, a neutron multiplier material Be-W alloy (the melting point is about 2023 K) was prepared by HIP. Beryllium and beryllium alloy (Be_12_Ti and Be_12_W) pebbles were successfully fabricated by a rotating electrode process. The irradiation blistering and swelling behavior of the beryllium and beryllium alloy sample were evaluated by high-dose helium irradiation. The mechanism of blistering and gas bubble nucleation and growth has been investigated and discussed.

## 2. Experimental Procedure

Beryllium, beryllium tungsten alloy, and beryllium titanium alloy pebbles were analyzed in this study, which were fabricated by HIP and a rotating electrode process (REP) from Baoji Haibao Special Metal Materials Co., Baoji, China. Beryllium metal powder (63 wt.% Be) and tungsten powder (37 wt.% W) were mixed, which is the stoichiometric composition of Be_12_W, loaded into a package sheathing, punched and seal welded. Then, the Be-W intermetallic compounds were synthesized by HIP with a pressure of 230 MPa at 1453 K for 4 h. The fabrication methods of beryllium and beryllide pebbles are presented in [29]. The phase composition of the beryllide (Be-Ti and Be-W) pebbles fabricated via the REP was evaluated by X-ray diffraction. According to the XRD results, the structure of Be-Ti sample contains Be_12_Ti phase with a tetragonal lattice and small Be phase with hexagonal close-packed lattice, and the structure of Be-W sample contains Be_12_W phase with a tetragonal lattice and small Be and W phase. The microsphere surface of irradiated and unirradiated beryllium and beryllide pebble samples was investigated using a Zeiss Auriga scanning electron microscope (SEM) (ZEISS, Oberkochen, Germany). Resolution of this SEM is up to 1.0 nm @ 15 kV. The detection range of elements is from Be (4) to Fm (100). Microstructure characterization of the specimens was carried out using (scanning) transmission electron microscopes (S/TEM: Tecnai F20) (FEI company, Hillsboro, OR, USA). This TEM was equipped with a high-angle annular dark field (HAADF) detector and an energy dispersive X-ray spectrum (EDS) analysis system. The accelerator voltage of this microscope was 200 kV.

**Figure 1:**
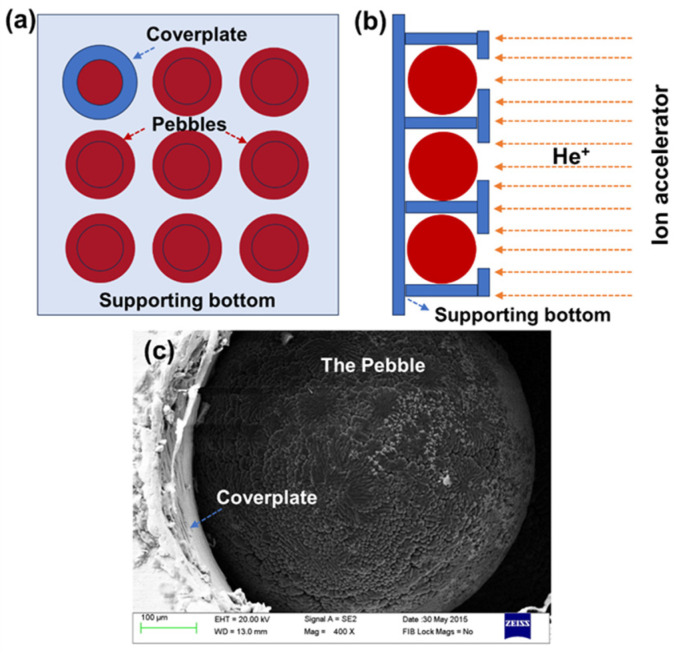
Scheme of pebble sample holder (**a**), the irradiation process (**b**) and a real photo of the holder and the pebble sample (**c**).

**Figure 2:**
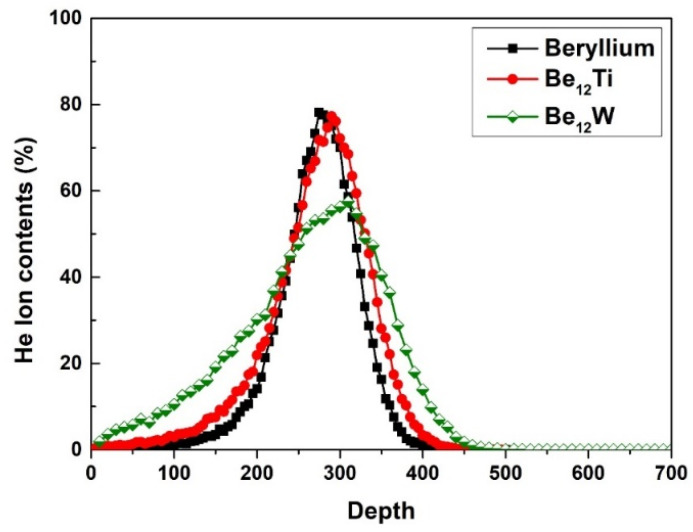
Helium content profile in the beryllium and beryllide samples calculated by SRIM.

The beryllium and beryllide samples were affixed in a homemade sample holder and then irradiated by helium ions using an ion accelerator. The scheme of the pebble sample holder and the irradiation process is shown in Figure 1. Figure 1c shows an SEM photo of the holder and pebbles. Before ion irradiation, the pebbles were placed on a pure-Cu supporting bottom. Then, the pebbles were fixed by a cover-plate with small holes (diameter is 0.5 mm). Next, 30 keV helium ions with an ion fluence of 1 × 10^18^ He^+^/cm^2^ were implanted perpendicularly into the surface of the sample through the small holes in the cover plate at room temperature. Helium ions content in the pebble specimen was calculated by the Monte Carlo program SRIM 2013 and a calculation script [30,31]. The peak helium content was about 78.2 at.% in beryllium, 77.3 at.% in Be_12_Ti and 57.5% at.% in Be_12_W based on the calculation as shown in Figure 2. The implanted helium has more concentrated distribution in Be and Be_12_Ti in comparison to that of Be_12_W.

## 3. Results and Discussion

**Figure 3:**
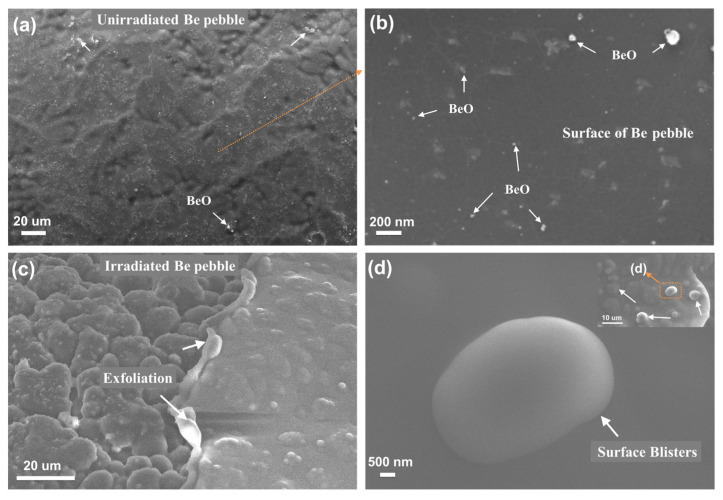
Surface microstructure of unirradiated (**a**,**b**) and irradiated (**c**,**d**) Be pebble.

**Figure 4:**
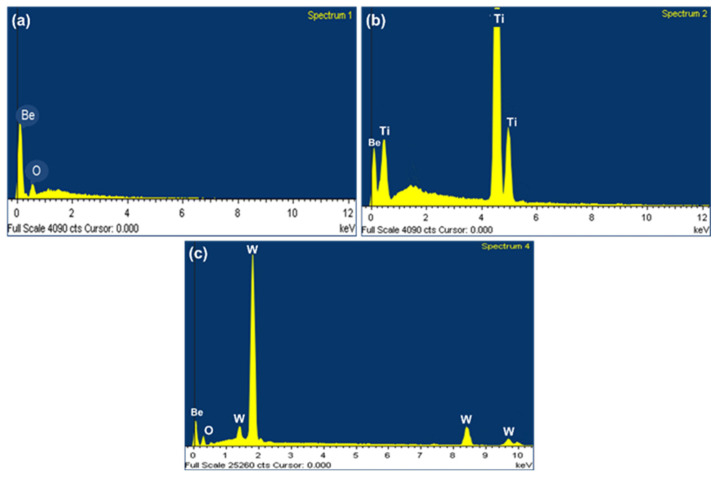
The EDX spectrums of Be (**a**), Be-Ti (**b**) and Be-W (**c**) pebbles.

Figure 3 shows the surface microstructure of unirradiated and irradiated beryllium pebble fabricated by REP. The beryllium pebble is well shaped with a metallic sheen, as shown in Figure 3a. EDX spectra of Be, Be-Ti and Be-W pebbles are shown in Figure 4. According to the EDX analysis, the content of beryllium in the pebbles is 98.1%, which can meet the design need of TBM [32]. There are some BeO impurities on the surface of beryllium pebbles, as shown in Figure 3a,b. After being irradiated by high-dose helium ion, surface blistering and even exfoliation were observed, as shown in Figure 3c,d. The surface bubbles are distributed uniformly and its average size is about 5.5 µm.

**Figure 5:**
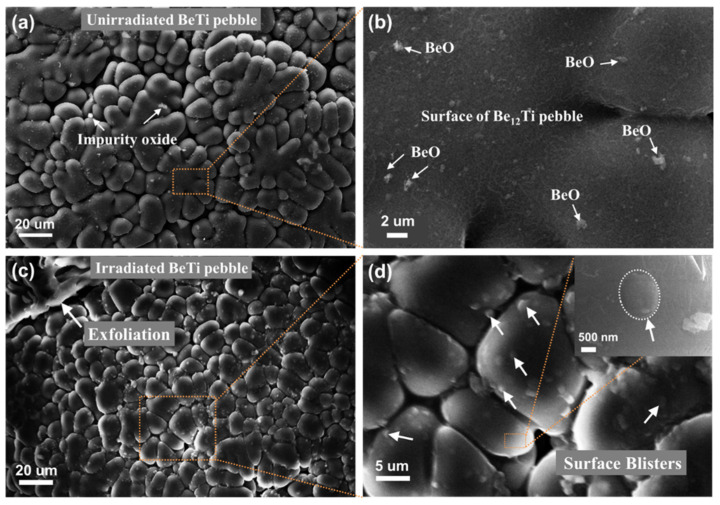
Surface microstructure of Be_12_Ti pebble before (**a**,**b**) and after ion irradiation (**c**,**d**).

Figure 5a,b show the surface microstructure of the Be-Ti pebble. Be-Ti pebbles are also in good shape, with sphericity greater than 90%. Dendritic structure and grain boundaries of the Be_12_Ti grains are clearly observed. The surface is smooth without obvious bulges. There are also some BeO impurities on the surface of Be-Ti pebbles, as shown in Figure 5b. After irradiation by high-dose helium ion, surface blistering and exfoliation were also observed, as shown in Figure 5c,d. In comparison to the beryllium pebble, fewer exfoliation areas were observed; in addition, the surface bubbles were smaller with an average size of about 1.1 µm.

Combining with the XRD, SEM and EDS results, the gray-black region is found to be Be_12_W phase (with about 62.9 wt.% W) and a small local gray-white region is single-W phase. The Be-W alloy prepared by HIP method has a gray and shiny surface, as reported in [33]. In order to obtain a single Be_12_W phase material, subsequent homogenization treatment could be used. Nakamichi and Kim et al. reported the preparation and homogenization treatment of Be_12_Ti alloy [26,34,35,36]. Although a single phase can be obtained by homogenization, the porosity of the material will increase and the reaction of the materials with water will also increase with more hydrogen generated. In this work, the Be-W alloy has not been homogenized. After irradiation by high-dose helium ion, surface blistering was observed, but no exfoliation was observed. Some surface bubbles were larger in size, while some surface bubbles were smaller. The size difference between the large (with the average size about 0.8 µm) and small bubble (with the average size about 79 nm) was quite large, with the small bubble always forming on the large bubble. There were also some large bubbles that burst in the local area.

**Figure 6:**
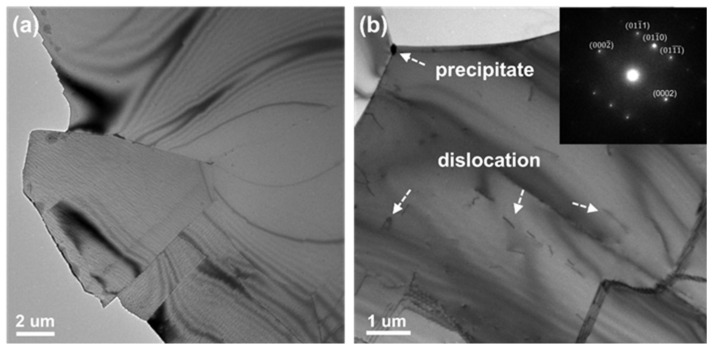
Microstructure of Beryllium before ion irradiation. The brightfield image (**a**) and two-beam dynamic brightfield image (**b**).

TEM and STEM analysis was carried out to confirm the internal microstructure of the beryllium specimen with and without the ion irradiation. Figure 6 shows the brightfield image (a) and two-beam dynamic brightfield image (b), with $g=10\bar{1}1$ of unirradiated beryllium sample. Beryllium grains vary in shape and size. The grain size of the beryllium specimen ranges from several microns (about 8.2 µm as shown in Figure 6a) to several hundred microns. Dislocation lines with low density were observed in the sample, as shown in Figure 6b, which could be induced during the manufacturing process. The electron diffraction pattern (EDP) of the corresponding grain was shown in the inset of Figure 6b. There are no extra diffraction spots except for the matrix ones in the EDP, which confirms that the tiny, segmented substance that appeared in Figure 6b is not precipitates but dislocation lines. Particularly, there are regions that are free from dislocations. Some impurity particles or precipitates in the matrix or near the grain boundary were observed, also as shown in the morphology.

**Figure 7:**
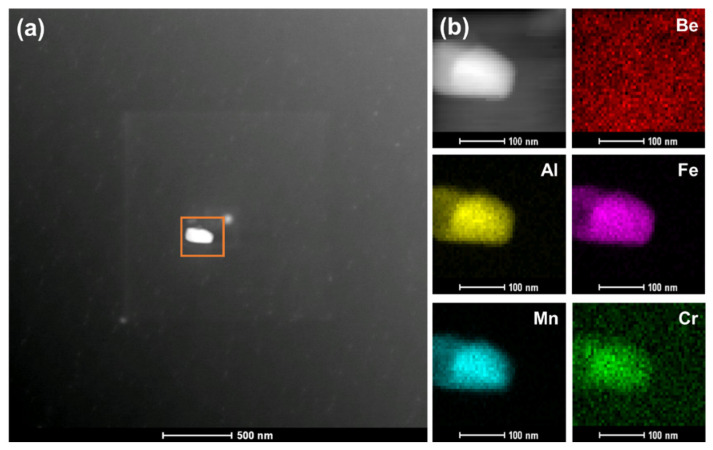
The HAADF image and EDS mapping of a precipitate in unirradiated beryllium sample. (**a**) The low-magnification Z-contrast image; (**b**) the composition mapping of the precipitate.

The high-angle annular dark-field (HAADF) and STEM analysis were performed to understand these precipitates more clearly. Figure 7a shows a typical low-magnification Z-contrast (Z is the atomic number) imaging of the beryllium sample. Z-contrast imaging can help eliminate the contrast contributions from coherent strain effects and highlight the mass-thickness differences. STEM-EDS mapping images of the area marked by an orange square in Figure 7a are shown in Figure 7b. The mapping images are shown according to the K-edge of the Be/Al/Fe/Mn/Cr elements. According to the EDS, this precipitate with rectangle shape shows a complex composition of 36.25 wt.% Al, 23.16 wt.% Fe, 16.23 wt.% Mn and 10.76 wt.% Cr. In combination with the electron diffraction pattern of the precipitate, it should be Be_4_Al (Fe, Mn, Cr) phase with an FCC structure. The precipitate exhibits brighter contrasts than those of the beryllium matrix because of the enrichment of heavy atoms of Fe, Al, Mn and Cr.

**Figure 8:**
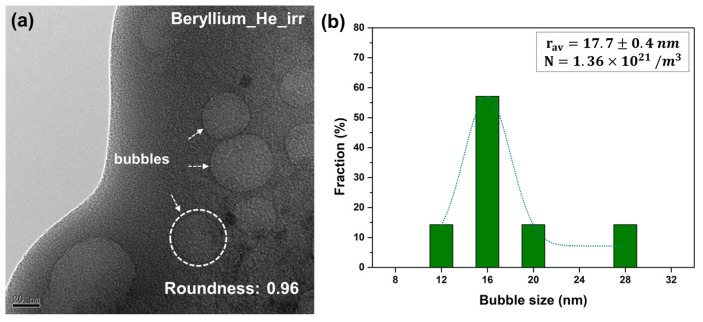
(**a**) Large bubbles in beryllium irradiated by high dose helium ion irradiation; (**b**) Size distribution and number density of helium bubble after high dose helium ion irradiation.

When the sample was irradiated with high-dose helium ions at room temperature, a large number of helium bubbles were generated inside the sample. A number of large bubbles (size of ~30 nm) were observed in the beryllium sample irradiated by the 1 × 10^18^ He^+^/cm^2^ helium ion irradiation, as shown in Figure 8a. The roundness of the He bubbles in Be after irradiation was measured about 0.96, which indicated they should be spherical bubbles. Figure 8b shows the radius distribution, average radius and number density of the He bubbles. The size of the bubble is from 11 nm to 28 nm, with an average radius of 17.7 ± 0.4 nm. The number density is about 1.36 × 10^21^/m^3^. An effective swelling rate S of materials can be calculated according to the increased volume of materials, which is equal to the sum of all helium bubbles’ volume, corresponding to the volume. The swelling rate was calculated to be about 3.7%.

There are two possible mechanisms of gas (i.e., He) bubble nucleation at low temperature (T < 0.2 Tm, and Tm is melting point). Under the premise that the vacancies caused by thermal vacancies and irradiation can be ignored, the “di-atomic nucleation” model assumes that two helium atoms in the matrix will attract each other to form a stable nucleus [37,38,39]. According to the “di-atomic nucleation” model, helium bubble formation and growth are mainly controlled by the diffusion of helium atoms. Recently, molecular dynamics simulations show that the binding energy of two helium atoms (the distance between two atoms is 1.74 Å) forming the stable nucleus was 0.63 eV, and it increases with the addition of more helium atoms [40]. This means that helium atoms are able to nucleate by trapping themselves, and that once a cluster of helium atoms has formed, it is difficult to decompose them. This is consistent with the di-atomic nucleation model at low temperature. When a large number of vacancies and interstitial atoms are produced by the irradiation, they will promote the aggregation of helium atoms and play a major role in growth of helium bubbles [41]. The results of molecular dynamics calculations show that interstitial atoms and vacancies can promote the aggregation of helium atoms, especially for the vacancies. The helium atom will occupy the center of the vacancy. In the presence of vacancies and interstitial atoms, helium atoms and vacancies (V) or interstitial atoms (I) can first form complexes (He–V and He–I), and then these complexes continue to trap vacancies and/or helium atoms, resulting in helium bubble formation and growth. In the nucleation and growth of helium bubbles, the above two mechanisms may both exist. The shape (roundness) of the helium bubble is usually affected by the air pressure inside the bubble. It is assumed that He behaves as an ideal gas, and the internal pressure of the bubble can be determined by [42]:

(1) $$PV=n_{He}k_{B}T$$

where *V* is the volume of the spherical bubble, $n_{He}$ is the amount of helium in the bubble, $k_{B}$ is Boltzmann’s constant, and *T* is the temperature. At high gas densities, the ideal gas equation of state becomes inaccurate. Then, the hard sphere equation of the state can be used as [42]:

(2) $$\frac{PV}{n_{He}k_{B}T}=\frac{\left(1+y+y^{2}-y^{3}\right)}{{\left(1-y\right)}^{3}}$$

with

(3) $$y=\frac{\pi n_{He}d_{g}^{3}}{6V}$$

where $d_{g}$ is the hard sphere diameter of the gas atoms as a function of temperature [42,43]. According to the SRIM calculation, the average density of helium is about 1.999 × 10^28^ ion/m^3^, and the average injection content in 500 nm depth is about 16.2 at% in beryllium sample with 1 × 10^18^ ion/cm^2^ He (30 keV) irradiation. Here, if we assume that the injected helium is evenly distributed in bubbles and the matrix, the average amount of helium in each bubble is calculated to be about = 4.64 × 10^5^. Combining Equations (1) and (2), the internal pressure of the bubble (*P*) is up to 0.14 GPa. Surface tension of the bubble is about 0.198 GPa. Then, the bubble should be partially faced. It is suggested that most of the helium enters the bubbles. Then, if we assume that all the helium is injected into the bubble, the average amount of helium in each bubble is calculated to be about =1.47 × 10^7^. Combining Equations (1) and (2), the internal pressure of the bubble (*P*) is up to 4.28 GPa. It is worth noting that this is the maximum value of the air pressure because the injected helium could not all go into the bubble due to the trapping of grain boundary or other interfaces in the materials. The high air pressure is the reason why the bubbles remain spherical with an average roundness of >0.92.

**Figure 9:**
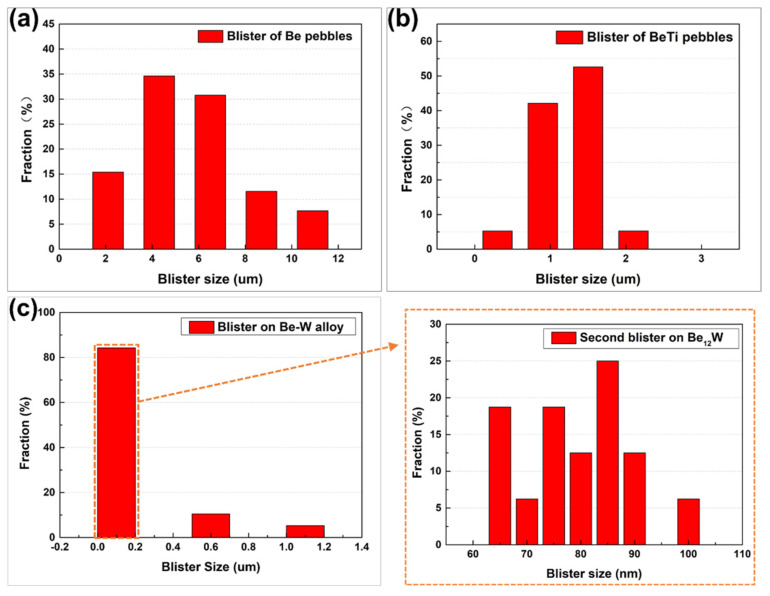
Bubble size and density of the beryllium (**a**) and beryllide sample (**b**) for Be-Ti and (**c**) for Be-W) after helium ions irradiation.

**Figure 10:**
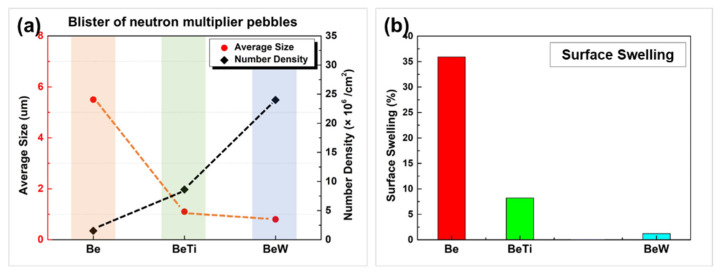
Bubble size distribution (**a**) and the surface swelling (**b**) of the beryllium and beryllide sample after helium ions irradiation.

With the continuous injection of helium ions, new helium bubbles may be generated and the original helium bubbles grow by absorbing helium atoms, vacancies and/or He–V complexes. But because the distribution of ion implantation is uneven (as shown in Figure 2) and the defect distribution is similarly uneven, a large bubble band will be generated underneath the surface of the sample. Evans [44] proposed a blistering mechanism explained by interbubble fracture, with a formula about the stress s between bubbles as follows,

(4) $$\sigma =P^{\prime }\pi r^{2}/\left(d^{2}-\pi r^{2}\right)$$

and

(5) $$P^{\prime }=P-2\gamma /r$$

where $P^{\prime }$ is an excess internal pressure in a gas bubble, *r* is radius of the bubble, d means the distance between nearest neighbor centers, *P* is the internal pressure of the bubble, and *γ* is the surface tension. Here, the 2*γ*/*r* is calculated to be about 0.198 GPa. Then, the excess pressure ($P^{\prime }$) should <4.08 GPa. It is worth noting that this is the average value. In the injection peak area, the excess pressure will be larger. When the internal pressure and excess pressure are large enough for σ to exceed the yield strength of the material, some large bubbles could be merged into a huge bubble (or internal crack) several micrometers in diameter and resulting in a surface blister. With the continuous bombardment of ions and the continuous increase in air pressure inside the bubbles, some large bubbles may also burst and strip off. If the ion implantation dose is large enough, the subsequent generations of blisters (called “secondary blisters”) will form in the post-exfoliated surface [31]. In this study, obvious second generations of blisters were observed in beryllium and beryllide samples. The statistical data of the surface bubble size and density after helium ion irradiation are shown in Figure 9 and Figure 10. In comparison to Be and Be-Ti pebbles, the average size of secondary bubbles is very small (<100 nm) and no exfoliation was observed.

In order to preliminarily quantify the anti-irradiation blistering properties of the three materials, the surface blistering rate (surface swelling rate) was used here:

(6) $$s\_swelling=\frac{\sum s\_bubble}{S_{0}}\times \%$$

where $S_{0}$ is the original area of the irradiated area and ∑s_bubble is the sum area of all bubbles in the irradiated area. The calculated results are shown in Figure 10b. The surface swelling rate of beryllium pebbles is up to 35% (red marked in the histogram), and that of Be-Ti pebbles is about 8% (green marked in the histogram), while the surface swelling rate of Be-W pebbles is about 1.2% (blue marked in the histogram). This result may underestimate the irradiation swelling resistance of Be_12_W alloy to low-temperature helium ion because most bubbles were observed on the W phase, not on the Be_12_W phase in Be-W alloy. The preliminary blistering mechanism based on the Evans equation [44] has been discussed in the previous part. However, the helium trapping position, binding energy and activation energy of helium in beryllide, especially Be_12_W, need to be studied further.

## 4. Conclusions

In this study, Be-W alloys mainly composed of Be_12_W phase were fabricated by HIP, which may be a candidate for neutron multiplier material because of its high melting point and high beryllium content. Beryllium and beryllide (Be_12_Ti and Be_12_W) samples were irradiated by helium ion with 30 keV and 1 × 10^18^ cm^−2^ at RT. The microstructures of Be, Be_12_Ti and Be_12_W samples were analyzed before and after ion irradiation. After high-dose helium ion irradiation, surface blistering and even secondary blistering were observed on all three kinds of samples. The average size of the first blistering on the surface of Be-W alloy is about 0.8 μm, and the density is about 2.4 × 10^7^ cm^−2^, while the average size of the secondary blistering is about 80 nm and the density is about 1.28 × 10^8^ cm^−2^. Exfoliation was observed on the Be and Be_12_Ti samples, but not on the Be_12_W sample. The microstructure (i.e., dislocation, precipitate and bubble) of the beryllium was characterized using TEM and STEM. The internal pressure of the bubbles in the beryllium was first estimated. It was found that the pressure was very high, which may be the reason for the highly round spherical bubble and the surface blistering. The surface blistering rates of the beryllium and beryllide samples were also compared. These results indicate that the Be_12_W alloy may have high irradiation swelling resistance under high-dose helium irradiation.

## Data Availability

Data is contained within the article.
